# Observations on comatose survivors of cardiopulmonary resuscitation with generalized myoclonus

**DOI:** 10.1186/1471-2377-5-14

**Published:** 2005-07-18

**Authors:** Frank Thömke, Jürgen J Marx, Oliver Sauer, Thomas Hundsberger, Stefan Hägele, Jascha Wiechelt, Sacha L Weilemann

**Affiliations:** 1 Department of Neurology, Johannes Gutenberg-Universität, Langenbeckstrasse 1, D- 55101 Mainz, Germany; 2 Internal Medicine II, Johannes Gutenberg-Universität, Langenbeckstrasse 1, D- 55101 Mainz, Germany

## Abstract

**Background:**

There is only limited data on improvements of critical medical care is resulting in a better outcome of comatose survivors of cardiopulmonary resuscitation (CPR) with generalized myoclonus. There is also a paucity of data on the temporal dynamics of electroenephalographic (EEG) abnormalities in these patients.

**Methods:**

Serial EEG examinations were done in 50 comatose survivors of CPR with generalized myoclonus seen over an 8 years period.

**Results:**

Generalized myoclonus occurred within 24 hours after CPR. It was associated with burst-suppression EEG (n = 42), continuous generalized epileptiform discharges (n = 5), alpha-coma-EEG (n = 52), and low amplitude (10 μV <) recording (n = 1). Except in 3 patients, these EEG-patterns were followed by another of these always nonreactive patterns within one day, mainly alpha-coma-EEG (n = 10) and continuous generalized epileptiform discharges (n = 9). Serial recordings disclosed a variety of EEG-sequences composed of these EEG-patterns, finally leading to isoelectric or flat recordings. Forty-five patients died within 2 weeks, 5 patients survived and remained in a permanent vegetative state.

**Conclusion:**

Generalized myoclonus in comatose survivors of CPR still implies a poor outcome despite advances in critical care medicine. Anticonvulsive drugs are usually ineffective. All postanoxic EEG-patterns are transient and followed by a variety of EEG sequences composed of different EEG patterns, each of which is recognized as an unfavourable sign. Different EEG-patterns in anoxic encephalopathy may reflect different forms of neocortical dysfunction, which occur at different stages of a dynamic process finally leading to severe neuronal loss.

## Background

Comatose survivors of cardiopulmonary resuscitation (CPR) developing generalized myoclonus have a poor prognosis. Most of these patient die and those surviving the acute stage almost always remain in a persistent vegetative state [1-8]. There are recent reports of single patients with myoclonus who have a good outcome [9,10], suggesting poor prognosis is not invariably the case. There is only limited data on whether advances in critical care medicine are associated with a better outcome. We address this issue on the basis of 50 comatose survivors of CPR with generalized myoclonus, whom we examined and treated during the past 8 years. We also report the results of serial electroencephalographic (EEG) recordings, there being only limited data on the temporal dynamics of EEG abnormalities in the acute stage after CPR. Finally, we discuss the relation of generalized myoclonus and status epilepticus, since terms, such as "*myoclonic status epilepticus" *[4,5], *"generalized status myoclonicus" *[2], and *"myoclonus status" *[6], imply that comatose survivors of CPR with generalized myoclonus suffer from a severe form of convulsive status epilepticus.

## Methods

Over an 8-year-period (between winter 1996/1997 and winter 2004/2005), we observed 50 consecutive patients, who developed generalized myoclonus within 24 hours after CPR. There were 24 women, aged 26 to 83 years, mean age: 55 years; and 26 men, aged 20 to 79 years, mean age 53 years. Forty-five patients were resuscitated outside and the remaining 5 inside the hospital. Forty-four patients had cardiac arrest or ventricular fibrillation, 4 acute respiratory failure, and 2 circulatory collapse due to gastrointestinal bleeding.

All patients had bipolar 8-channel EEG recordings with needle electrodes positioned according to the standard 10–20 system of electrode placement (Fp2-T4, T4-O2, Fp2-C4, C4-O2, Fp1-T3, T3-O1, Fp1-C3, C3-O2.). Filter setting was 0.53 Hz and 70 Hz. The first EEG was done 6 to 24 hours after CPR in all 50 patients. At that time, all had generalized myoclonus and none were on sufficiently high doses of drugs that would produce a burst-suppression pattern: Approximately 0.2 mg midazolam/kg body weight and 0.5 mg fentanyl were usually given when the patients were admitted to the intensive care unit. Subsequent EEGs were recorded in survivors at day 2 (42 patients), at day 3 or 4 (29 patients), at day 5 or 6 (15 patients), at 7 or 8 (12 patients), and during the 2^nd ^week (4 patients). Several medications (phenytoin, valproic acid, diazepam, clonanzepam, lorazepam, midazolam, propofol) were used in an attempt to suppress generalized myoclonus after the first EEG. EEGs at day 2 were usually done after the administration of one these drugs given in doses usually used for convulsive status epilepticus, i.e. phenytoin: 1500 mg within 30 to 60 minutes; valproate: 1600 to 3200 mg within 30 minutes; diazepam up to 40 mg, clonazepam or lorazepam up to 8 mg, or midazolam up to 15 mg.

Diagnosis of a burst-suppression EEG (BS-EEG) was based on the *Guidelines of the International Federation of Clinical Neurophysiology*: "bursts of theta and/or delta waves, at times intermixed with faster waves, and intervening periods of low amplitude (below 20 μV)" [11], and on *Niedermeyer and Lopes da Silva's Electroencephalography*: "high-voltage bursts of slow waves with intermingled sharp transients or spikes occur against a depressed background or complete flatness" [12]. In accordance with others [13-15], we fixed the duration of isoelectric or low amplitude interburst intervals to at least 1 second to exclude patients with generalized continuous epileptiform discharges. Flat recordings were those with amplitudes below 20 μV, and isoelectric recordings those without any detectable activity (sensitivity of the recording system: 2 μV/mm). Serial determinations of serum neuron-specific enolase (NSE) were done one, two and three days after CPR in 27 of the 50 patients.

## Results

All patients with generalized myoclonus were comatose, needed mechanical ventilation, and had loss of some brainstem reflexes. Myoclonus was highly variable ranging from single myoclonic jerks to nearly continuous myoclonus. It was generalized in approximately two thirds of patients and multifocal in one third. In all patients, myoclonus involved the facial muscles, more often associated with bilateral eye closure than with bilateral eye opening. Occasionally, myoclonus was restricted to facial muscles and manifested as eye or jaw opening. Most patients also had myoclonus of the shoulder and proximal arm muscles and the diaphragm. Involvement of the legs occurred less often but was seen in approximately half of the patients. Myoclonus almost always increased or was triggered by acoustic stimuli, touch, and tracheal suctioning.

In general, intravenous phenytoin, valproate, or various benzodiazepines were ineffective when given in doses usually used in patients with convulsive status epilepticus, i.e. phenytoin: 1500 mg over 30 to 60 minutes; valproate: 1600 to 3200 mg over 30 minutes; diazepam up to 40 mg, clonanzepam or lorazepam up to 8 mg, or midazolam up to 15 mg. Intravenous propofol (100 to 300 mg) was given to the last 7 patients after the recording of the 2^nd ^EEG. This was always followed by a flat (below 10 μV) EEG recordings and cessation of generalized myoclonus, the cessation persisting during continuous propofol infusion (150 to 250 mg/h). Except in one patient whose generalized myoclonus persisted with decreasing intensity until her death 9 days after CPR, generalized myoclonus usually ceased within 1 to 2, but occasionally 3 days.

**Figure 1:**
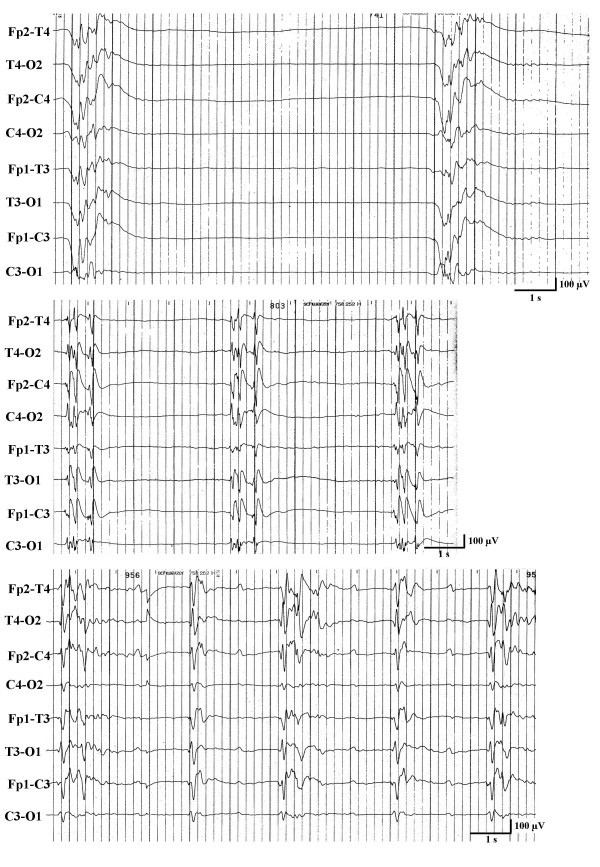
Three examples of a burst-suppression-EEG of 3 comatose survivors with generalized myoclonus within 24 hours after cardiopulmonary resuscitation.

**Figure 2:**
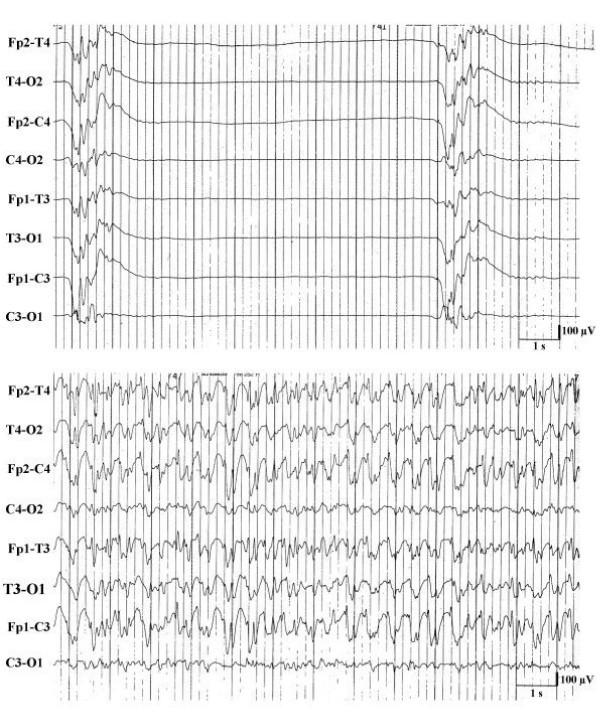
Burst-suppression-EEG (upper recording) with periods of continuous epileptiform discharges (lower recording) in a comatose survivors of cardiopulmonary resuscitation with generalized myoclonus.

**Figure 3:**
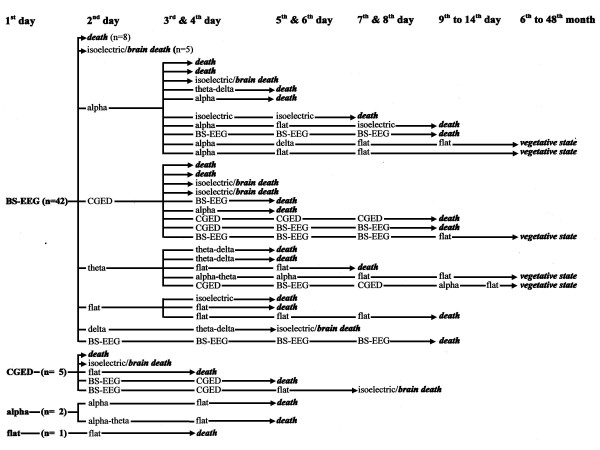
EEG-sequences and outcome in 50 comatose survivors of cardiopulmonary resuscitation with generalized myoclonus.

**Figure 4:**
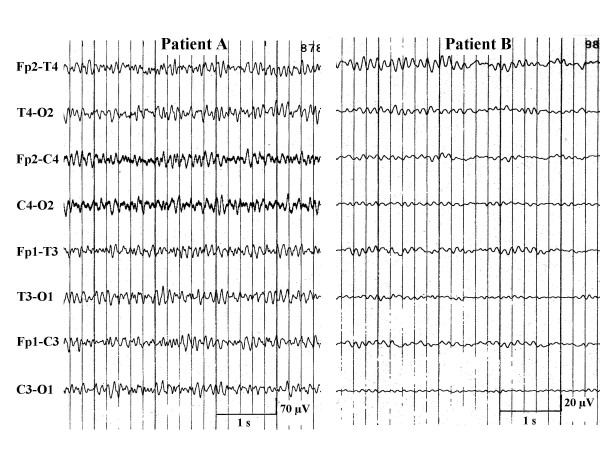
Examples of alpha-coma-EEGs on the 2^nd ^day after cardiopulmonary resuscitation in 2 comatose survivors with burst-suppression EEG on the preceding day.

**Figure 5:**
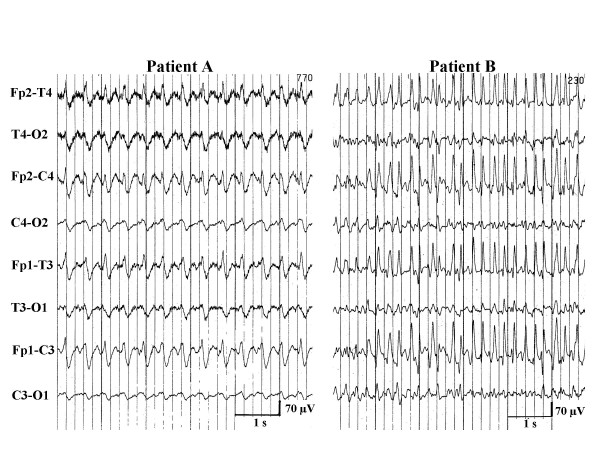
Examples of continuous epileptiform discharges on the 2^nd ^day after cardiopulmonary resuscitation in 2 comatose survivors with burst-suppression EEG on the preceding day.

**Figure 6:**
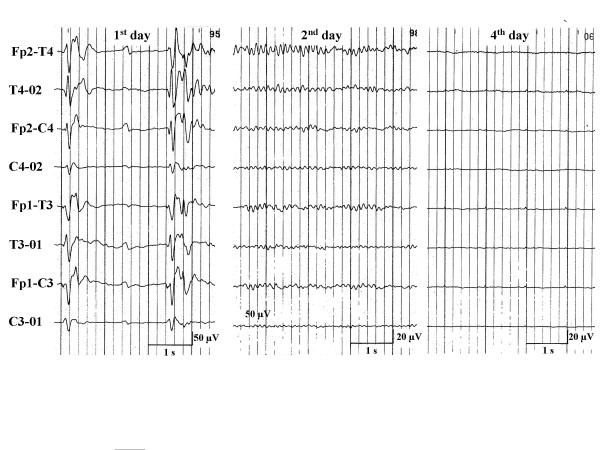
EEG-sequence of a comatose survivors of cardiopulmonary resuscitation with a burst-suppression-EEG on the 1^st ^day, which was followed by an alpha-coma-EEG on the 2^nd ^and an isolelectric recording on the 4^th ^day.

**Figure 7:**
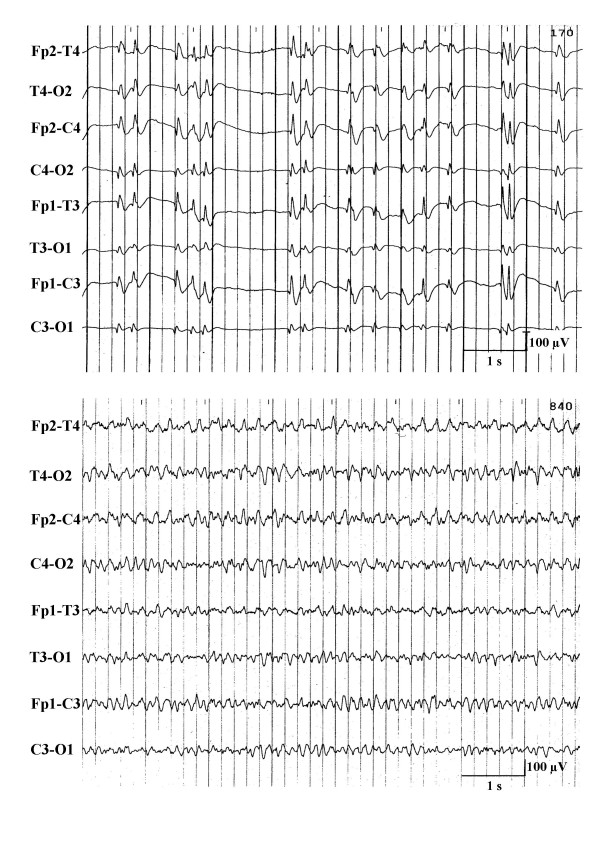
"Transitional"-EEG-pattern between burst-suppression-EEG and continuous epileptiform discharges (upper recording) or between alpha-and theta-coma EEG (lower recording).

In 42 patients, generalized myoclonus was associated with a BS-EEG (Figure 1), which, in 12 patients was interrupted by trains of continuous epileptiform discharges for 10 to 55 seconds (Figure 2). Burst and trains of continuous epileptiform discharges were usually associated with generalized myoclonus. Sometimes bursts of activity without any visible myoclonus were recorded and at other times myoclonic jerks were seen without associated bursts. In the remaining 8 patients, generalized myoclonus was associated with continuous generalized epileptiform discharges (n = 5), alpha-coma-EEG (n = 2), and a low amplitude (below 10 μV) recording (n = 1), i.e. without associated EEG bursts. Except in one patient, who had a BS-EEG until her death 9 days after CPR, BS-EEG and any other EEG-pattern were only a transient phenomenon always followed within one day by another nonreactive EEG pattern (Figure 3). The most frequent nonreactive EEG-patterns on the 2^nd ^day were alpha-coma-EEG (with or without some theta activity) (n = 10) (Figure 4) generalized continuous epileptiform discharges (n = 9), (Figure 5), theta EEG (n = 5), or isoelectric recordings (accompanied by clinical signs of brain death, n = 5). Subsequent recordings disclosed highly variable EEG sequences characterized by transitions between these patterns including reappearance of a BS-EEG and, finally, a low amplitude or isoelectric recording (Figures 3 and 6). Transitions between different EEG patterns were often subtle, especially transitions between between BS-EEG and continuous epileptiform discharges or between alpha-and theta-coma-EEG (Figure 7). Coexistence of different EEG-patterns in the same recording was seen in 18 patients, mainly BS-EEG with trains of generalized continuous epileptiform discharges (between 10 and 55 seconds) in 12 patients (Figure 2). Other coexisting patterns included alpha-coma-EEG with stretches of epileptiform discharges (up to 10 s) (n = 1) or with intervening periods of low amplitude (for 2 s) (n = 1), generalized continuous epileptiform discharges with trains of alpha-theta-acticity (for 2–5 s) (n = 2), theta-EEG with bursts of epileptiform discharges (for 1–3 s) (n = 1), BS-EEG with trains of alpha-theta-activity (for 2–4 s) (n = 1), and BS-EEG with episodes of alpha (theta) activity and periods of generalized continuous epileptiform discharges (n = 1).

Serum NSE was elevated in all 27 patients with maximum values between 36 und 540 ng/ml (upper normal limit in our laboratory: 17 ng/ml). Fifteen patients died within 24 hours and another 9 within 4 days after the resuscitation. Determinations regarding the level of care to be provided were made 3 to 4 days after CPR in 26 patients surviving up to this point. If clinical examination, EEG, and serum-NSE levels indicated a poor prognosis, treatment was restricted to mechanical ventilation and intravenous fluids. At the time of this decision, each patient had (a) no motor response on painful stimuli, (b) loss of the vestibular-ocular reflex, (c) NSE-levels between 67 and 540 ng/ml, and (d) three recordings of an unfavourable EEG pattern, i.e. burst-suppression EEG, continuous epileptiform discharges, or alpha-coma-EEG. Moreover, about one third also had bilateral absence of the pupil light reaction and/or bilateral absence of the corneal reflex. Twenty-one of these patients died between the 5^th ^and 12^th ^days after the resuscitation. None needed mechanical ventilation for more than 7 day. The remaining five patients survived in a persistent vegetative state.

## Discussion

Two different forms of myoclonus may occur in patients with hypoxic injury of the brain. An acute form, the focus of the present paper, is different from the chronic form. The chronic form is seen in patients who regain consciousness and have the myoclonus begin days to weeks after CPR. Chronic hypoxic myoclonus is an action myoclonus, which only occurs when the conscious patient moves his arm or leg and is restricted to the extremity being moved. This myoclonus is thought to be generated by the cerebral cortex and probably reflects impairment of serotonergic transmission [16]. These patients often have only a mild or moderate intellectual impairment and most also suffer from cerebellar ataxia [17-19]. For reasons of clarity, only these patients should be classified as "Lance-Adams syndrome".

The acute form seen in our patients occurs in comatose survivors within one day after CPR. It is characterized by generalized myoclonic jerks which occur spontaneously and increase with acoustic or tactile stimuli. This type of myoclonus is thought to be generated by brainstem structures, the hypoxic damage of the neocortex likely being too severe to generate myoclonus [16]. Generalized myoclonus in comatose survivors of CPR is a transient phenomenon and implies a poor prognosis. The majority of our patients died during the acute stage in less than 2 weeks, and the few survivors remained in a permanent vegetative state. This is in agreement with previous studies [1-8].

Improvement in the critical care of patients has not resulted in a better outcome (Table 1). All pathological studies in such patients disclosed severe and extensive neuronal loss in the cerebral cortex, basal ganglia, thalamus, cerebellar cortex and – when examined – also in the spinal cord [2,4-6]. Extensive neuronal loss is also reflected by the regular elevation of serum NSE in our patients, which, as an isolated phenomenon, is known to indicate a poor prognosis [20-25].

**Table 1:** Outcome of comatose survivors of cardiopulmonary resuscitation with generalized myoclonus in previous and the present series

**Study**	**No. of patients**	**died**	**persistent vegetative state**	**complete recovery**
***Butenuth and Kubicki *1971 [1]**	12	12		
***Celesia et al*. 1988 [2]**	13	8	4	1
***Krumholz et al*. 1988 [3]**	19	19		
***Jumao-as and Brenner *1990 [4]**	11	11		
***Young et al*. 1990 [5]**	15	15		
***Wijdicks et al*. 1994 [6]**	40	40		
***Reeves et al. 1997 *[8]**	9	9		
				
**this series**	50	45	5	

There are, however, reports on individual patients with a good outcome despite generalized myoclonus after CPR, but these reports leave some questions. Celesia et al. [2] mentioned complete recovery in one of 13 patients with generalized myoclonus, but their paper lacks any additional information, especially EEG-data. We were able to find another four more recently reported patients, all with status asthmaticus as the cause of cerebral hypoxia [9,10]. In these patients, myoclonus often occurred with reduction of benzodiazepines, and responded in all patients to anticonvulsant drugs. This would be very unusual for generalized myoclonus due to severe hypoxic injury of the brain and raises the question whether these patients may have suffered from epileptic myoclonus, i.e. myoclonic jerks as a symptom of epileptic seizures. As none of these patients had EEG recordings, this possibility cannot be excluded. At least in one of these patients, additional generalized tonic-clonic seizures were reported (case 1 in the paper of Morris et al. [10].

Epileptic myoclonus or convulsive status epilepticus need to be considered in another patient with anoxic encephalopathy due to ventricular fibrillation reported by Mori et al. [26]. This patient suffered from "uncontrollable generalized tonic-clonic convulsion" for about 24 hours followed by transient eye opening, which was synchronized with a burst-suppression EEG. The latter was controlled by 50 mg intravenous diazepam, an unusual finding in patients with generalized postanoxic myoclonus. This patients survived with "neurologic resisuduals, ie, parkinsinism, dementia, and seizures".

Both, Arnoldus and Lammers [9] and Morris et al. [10], cited another two previously reported patients from Harper and Wilkes [27] as examples of a good outcome despite generalized myoclonus. These patients, however, had action myoclonus. The fact, that both patients were no longer comatose after finishing sedation with benzodiazepines had escaped observation, and the correct diagnosis of multifocal action myoclonus was determined during the course of the illness [27]. Obviously, Harper and Wilkes were aware of the correct diagnosis as they entitled their paper "Posthypoxic myoclonus (the Lance-Adams syndrome) in the intensive care unit" [27], whereas Arnoldus and Lammers [9] and Morris et al. [10] likely misinterpreted clinical findings in these patients. Thus, we are not aware of any comatose survivor of CPR with generalized myoclonus and a good outcome when accompanied by a BS-EEG or generalized continuous epileptiform discharges.

As in to previous series, generalized myoclonus in comatose survivors of CPR was mainly associated with a BS-EEG, and occasionally with generalized continuous epileptiform discharges, alpha-coma-EEG or flat recordings [1,3,4,6-8]. Each of these EEG-patterns is recognized as an unfavourable sign [1-7,13,15,28-35]. Our data indicate that each of these EEG-pattern is a transient phenomenon in comatose survivors of CPR with generalized myoclonus. It is followed by another transient EEG-pattern from this group. The temporal EEG-dynamics after CPR is characterized by a variety of variable EEG-sequences composed of these unfavourable patterns, and finally resulting in isoelectric or flat recordings (figures 3 and 6). This confirms our own findings in a previously reported smaller group of patients with postanoxic BS-EEG [8]. Our more recent findings indicate the existence of similar temporal dynamics in comatose survivors of CPR with other, less frequently seen EEG-patterns (Figure 3). These data correspond to previous observations of Wijdicks et al. [6], who performed one repeat-EEG in nine patients with BS-EEG. In six patients, BS-EEG persisted, and three had transition to alpha-coma-EEG. They also performed repeated EEGs in one patient disclosing frequent alternations between BS-EEG and an alpha coma pattern [6]. This paper, however, lacked information on the time interval between BS-EEG and repeat-EEG, and we are not aware of any other study dealing with the temporal dynamics of postanoxic EEGs.

Nineteen of our 50 patients also showed coexistence of different unfavourable EEG-patterns in the same recording, mainly BS-EEG with trains of continuous epileptiform discharges (Figure 2), and occasionally alpha-coma-EEG with trains of epileptiform discharges or intervening periods of low amplitude, theta-coma-EEG with bursts of epileptiform discharges, and BS-EEG with episodes of alpha-theta-activity. Such a coexistence seems to be a frequent phenomenon occurring more often than suggested by previous observations in single patients with transitions between alpha-and theta-coma-EEG, and between BS-EEG and alpha-coma-EEG [6,36-40].

Different EEG-patterns and EEG-sequences in comatose survivors of CPR with generalized myoclonus probably reflect different forms of dysfunction of severely damaged neocortical neurons, and occurring at different stages of a dynamic process, finally leading to severe neuronal loss. BS-EEG and alpha-coma-EEG are the most frequent patterns and have been the subject of discussion in the literature. Alpha-coma-EEG was attributed to a deafferentiation of cerebral neurons with the amygdaloid nuclei (and other subcortical structures) functioning as the final pacemakers of electric brain activity [34]. This assumption implies a residual function of dying neocortical neurons to generate alpha-(and theta-)activity under the influence of the amygdaloid nuclei (or other subcortical structures) for a short time span. Deafferentiation of cortical neurons from cortical grey matter in patients undergoing frontal lobotomy may also cause a BS-pattern [41,42]. Although deafferentiation of morphologically intact cerebral cortex is different from neocortical neuronal damage in anoxic encephalopathy, there may be severe and permanent functional undercutting of the cortex with diffuse deafferentiation of cortical neurons after prolonged cardiopulmonary arrest [43]. This may cause disinhibition of certain neocortical neurons or complex neuronal networks, creating increased excitation. More recent findings also indicate that disconnection of brain circuits may be involved in the generation of BS-EEG [43]. These assumptions also imply some kind of preserved excitability of the dying cerebral neurons to generate bursts of activity.

The mechanism connecting BS-EEG with generalized myoclonus in comatose survivors of CPR has also been discussed in the literature. Since myoclonus and bursts occur fairly synchronously, it was suggested that excitation of excitation of motoneurons in the brainstem and spinal cord occurs as in epileptic seizures [7,43]. This would imply a coexistence of severe deafferentiation of neocortical neurons as a possible mechanism of BS-EEG with intact efferent pathways projecting tom brainstem and spinal cord motoneurons. There is, however, no satisfactory answer regarding whether severe deafferentiation can really coexist with still functioning efferent pathways (43). A number of previous observations as well as our own document bursts of EEG activity without any visible movements or the presence of myoclonic jerks without associated bursts [7,45], BS-EEGs with complex sequences of limb movements [7], or eye movements [46], or tonic posturing during the suppression phase [45]. This has led to the assumption that some of the motor phenomena accompanying BS-EEG are caused by a release of brainstem circuits. All in all, it seems, that different motor phenomena accompanying BS-EEG may be due to different mechanisms [7].

Generalized myoclonus in comatose survivors of CPR, also called "myoclonic status epilepticus" [4,5] or "generalized status myoclonicus" [2], was repeatedly discussed in association with convulsive status epilepticus, and classified as a subgroup of convulsive status epilepticus with a poor prognosis [47,48]. Comatose surviviors of CPR with generalized myoclonus, however, differ in some important aspects from patients with convulsive status epilepticus:

(a) Anticonvulsant drugs commonly used in the treatment of convulsive status epilepticus, i.e. intravenous phenytoin, valproate, or benzodiazepine, are usually ineffective in acute posthypoxic myoclconus [2-7,49], personal observation].

(b) The control of generalized myclonus by propofol, previously reported in single patients [49,50] and seen in seven of our patients, did not improve the prognosis of these patients although it resulted in the cessation of epileptiform discharges.

(c) EEG-patterns in patients with generalized myoclonus, especially BS-EEG and alpha-coma-EEG, differ fundamentally from those seen in convulsive status epilepticus [1-8].

(d) Posthypoxic generalized myoclonus is a self-limited phenomenon usually ceasing within 1 to 2 days and associated with severe and extensive neocortical neuronal loss [2,4-6]. Although convulsive status epilepticus – at least lethal status – may also cause neuronal loss, these anatomical alterations defects have a largely different distribution being maximal in the hippocampus, and are less severe then in anoxic encephalopathy [51]. In general, convulsive status epilepticus is not associated with severe neuronal loss and most survivors of one episode are without sequelae that impair cerebral function.

## Conclusion

Generalized myoclonus in comatose survivors after CPR still implies a poor prognosis despite improvement of the critical care of patients. It is a transient, self-limited phenomenon and reflects dysfunction of lethally damaged neurons [5]. As generalized myoclonus can be very disconcerting for relatives and staff, we recommend propofol, which often controls myoclonus (without improving the poor prognosis of these patients). Clinical, pathological, and EEG findings strongly indicate, that these patients die from severe hyoxic-ischemic damage rather than as a result of a "myoclonic status epilepticus". Abnormal EEG-patterns seen in these patients, especially BS-EEG and alpha-coma-EEG, may also be attributed to different forms of dysfunction of severely damaged neurons occurring at different stages of a dynamic process, finally leading to severe and extensive neuronal loss.

We determine the level of care to be provided 3 days after CPR. In patients with (i) unfavourable clinical signs (bilateral absence of the pupil light reaction and/or bilateral absence of the corneal reflex and/or loss of the vestibular-ocular reflex and/or no motor response on painful stimuli), (ii) an unfavourable EEG pattern (BS-EEG, generalized epileptiform discharges, alpha-coma-EEG), and (iii) elevated NSE serum levels, we restrict treatment to mechanical ventilation and intravenous fluids until they regain spontaneous breathing or die.

## Competing interests

The author(s) declare that they have no competing interests.

## Authors' contributions

FT conceived the study, examined all the patients, performed most of the EEG-recordings, participated in the analysis and interpretation of data, and drafted the manuscript

JJM participated in the analysis and interpretation of data, performed some EEG recordings, and helped to draft the manuscript

OS participated in the design of the study and in analysis and interpretation of data

TH participated in the analysis and interpretation of data and performed some of the EEG recordings

SH performed some of the EEG recordings and participated in the interpretation of data

JW participated in the design of the study and helped to draft the manuscript

SLW participated in the design of the study, helped to analyse the data and to draft the manuscript; and revised the manuscript critically

## Pre-publication history

The pre-publication history for this paper can be accessed here:


